# Avascular necrosis of femoral head following COVID-19 infection

**DOI:** 10.1097/MS9.0000000000001098

**Published:** 2023-07-25

**Authors:** Saywan K. Assad, Mohammed Sabah, Fahmi H. Kakamad, Abdulwahid M. Salih, Rawezh Q. Salih, Shvan H. Mohammed, Razhan K. Ali, Berun A. Abdalla, Marwan N. Hassan

**Affiliations:** a College of Medicine, University of Sulaimani; b Smart Health Tower; c Kscien Organization, Azadi Mall; d Shar Hospital, Department of Surgery, Sulaimani, Kurdistan, Iraq

**Keywords:** Avascular necrosis, Osteonecrosis, post-COVID-19 complications, SARS-CoV-2, Post-COVID-19 syndrome

## Abstract

**Introduction:**

It is crucial to be aware of post-COVID-19 non-pulmonary complications. Avascular necrosis (AVN) is one of these complications. It should be noted that the risk of AVN persists in individuals who have recovered from the COVID-19 infection. The current study aims to report several cases of AVN after being infected with SARS-CoV-2.

**Materials and methods:**

This is a single-centre retrospective case series conducted over a 2-year period (January 2021–December 2022) involving individuals who developed AVN after being infected with COVID-19.

**Result:**

The study included a total of 17 patients. The mean age of patients was 38.65±6.1 years. Twelve of them were male (70.6%) and five were female (29.4%), with a ratio of 3:1. The mean BMI of the patients was 28.3±2.4 kg/m^2^. Eleven (64.7%) patients reported administering steroid injections throughout the infection course. The mean interval between COVID-19 infection and presentation to the clinic was 6.53 months. The majority of patients (82.3%) complained of bilateral hip pain. Limping was observed in 47% of the cases. MRI showed AVN in all cases. Bilateral core decompression was performed in five cases (29.4%), total hip replacement in three cases (17.6%), and conservative treatment in nine cases (53%).

**Conclusion:**

The ongoing pandemic may have many long-term sequelae. There is a risk of developing AVN after COVID-19.

## Introduction

Highlights

- It is crucial to be aware of post-COVID-19 non-pulmonary complications.
- There is a risk of developing avascular necrosis after COVID-19.
- The current study aims to report several cases of avascular necrosis after being infected with SARS-CoV-2.

The COVID-19 emerged as the SARS-CoV-2, which was initially discovered in December 2019 in Wuhan, China. It rapidly expanded and developed into a pandemic illness^1,2^. SARS-CoV-2 binds more efficiently to the angiotensin-converting enzyme 2 receptor found in human glial cells, neurons, respiratory epithelial cells, and vascular endothelial cells^3^. Increasing evidence suggests that, while many patients are recovering from COVID-19, it has a negative impact on several human body systems as part of post-COVID-19 syndrome. The cardiopulmonary system appears to account for the majority of post-COVID-19 complications. Myocarditis, arrhythmia, and ischaemia are among the cardiac post-COVID-19 complications, whereas bacterial pneumonia, pneumothorax, and pleural effusion are among the most prevalent pulmonary complications^4^. It is crucial to be aware of non-pulmonary complications, such as strokes, renal failure, and peripheral arterial disorders^5^. Avascular necrosis (AVN) is one of these complications that, if ignored, can result in severe outcomes, including bone collapse. AVN was prevalent in SARS and may also be common in COVID-19 infections. It should be noted that the risk of AVN persists in individuals who have recovered from COVID-19 infection, similar to SARS^6^.

The current study aims to report several cases of symptomatic AVN of the femoral head after being infected with COVID-19.

## Materials and methods

### Study design

This is a single-centre retrospective case series study of patients who developed AVN after being infected with COVID-19. The ethical approval of the study was obtained from the ethical committee of the College of Medicine, University of Sulaimani. All patients provided written consent for their information to be published. The patients were assessed and treated over the past two years (January 2021–December 2022). The study has been written in line with PROCESS 2020 guidelines^7^.

All the references have been checked for reliability^8^.

### Participants

The sociodemographic and clinical data including age, sex, BMI, duration of COVID-19 infection, history of hospital or ICU admission, history of injection use, interval from the COVID-19 to the onset of symptoms, presentations, MRI, and management, were obtained from the patients, patients’ family, and patients’ medical records.

### Inclusion criteria

All patients with a prior history of COVID-19 who developed AVN were included in the study.

### Exclusion criteria

Patients with chronic or autoimmune diseases like rheumatoid arthritis, patients with a history of AVN prior to the COVID-19 infection, patients who lack covid-19 polymerase chain reaction evaluation, chronic alcoholic cases, and patients under chemotherapy for bone cancer.

## Result

The study included a total of 17 patients ranging in age from 21 to 60 years old (mean=38.65±6.1 years). Twelve of them were male (70.6%) and five were female (29.4%) with a ratio of 3: 1. The mean BMI of the patients was 28.3±2.4 kg/m^2^, with the majority falling within the range of 25–30 kg/m^2^ (47.1%). (Table 1). Eight (47%) patients had a history of hospitalization due to the COVID-19 infection, but none were admitted to the ICU. The mean duration of COVID-19 infection was 25.5 days, with a range of 12–52 days. Eleven (64.7%) patients reported receiving steroid injections during the course of infection, three (17.6%) did not, and three were unsure whether they had received the injections or not. The mean interval between COVID-19 infection and presentation to the clinic was 6.53 months, with a range of 2–13 months. Table 2 shows the demographic data and clinical characteristics of each case. The majority of patients (14, 82.3%) experienced bilateral hip pain, two (11.8%) had left hip pain and one (5.9%) had right hip pain. Limping was observed in eight (47%) of the cases, while the remaining portion (53%) had only pain. MRI revealed Grade 2 femoral head AVN in six sites, Grade 3 in twelve sites, Grade 4 in five sites, and the remaining eight sites were unclassified. Bilateral core decompression (CD) was performed in five cases (29.4%), total hip replacement in three cases (17.6%), and conservative treatment was administered in nine cases (53%) as they refused to undergo operation. Table 3 shows the site, presentation, MRI findings, and treatment plan for each of the patients.

**Table 1:** The baseline characteristics of the AVN cases

Variables	No. cases/frequency, *n* (%)
Demographics	
Age	
Third decade	5 (29.4)
Fourth decade	6 (35.3)
Fifth decade	2 (11.8)
Sixth decade	4 (23.5)
Mean age± SD (min–max)	38.65±6.1 (21–60 years)
Sex	
Male	12 (70.6)
Female	5 (29.4)
Anthropometric data	
BMI	
Normal	3 (17.6)
Overweight	8 (47.1)
Obesity	3 (17.6)
N/A	3 (17.6)
Mean BMI± SD (min–max)	28.3±2.4 (20.5–39) kg/m^2^
Medical history	
History of trauma	
Yes	8 (47)
No	9 (53)
History of bone disease	
No	17 (100)
Hospital admission during COVID-19	
Yes	8 (47)
No	9 (53)
Duration of hospital admission (day)	
12	1 (5.9)
15	4 (23.5)
16	1 (5.9)
20	2 (11.8)
21	1 (5.9)
30	5 (29.4)
38	1 (5.9)
52	1 (5.9)
N/A	1 (5.9)
Steroid use	
Yes	11 (64.7)
No	3 (17.6)
N/A	3 (17.6)
Duration of steroid use (day)	
7	3 (27.3)
5	4 (36.4)
4	3 (27.3)
2	1 (9)
Interval from COVID-19 and onset of symptoms (month)	
2–6	9 (53)
7–12	7 (41.1)
>12	1 (5.9)
AVN site	
Bilateral femoral head	14 (82.3)
Right femoral head	1 (5.9)
Left femoral head	2 (11.8)
Presentation	
Hip pain	17 (100)
Thigh Pain	2 (11.8)
Limping	8 (47)
Inability to walk	2 (11.8)

AVN, avascular necrosis; N/A, non-available.

**Table 2:** Demographic data and clinical characteristics of each case

No.	Age	Sex	BMI (kg/m^2^)	History of trauma	History of bone disease	Hospital admission during COVID-19	Duration of hospital admission (day)	ICU admission	Steroid use	Duration of steroid use (day)	Interval from COVID-19 and onset of symptoms (month)
1	36	Male	29	Yes	No	Yes	15	No	Unknown	Nil	3
2	33	Male	29	No	No	No	15	No	Yes	7	7
3	36	Male	26	No	No	No	15	No	Yes	7	4
4	59	Female	28.9	No	No	No	30	No	Unknown	Nil	3
5	23	Male	21	No	No	No	16	No	Yes	7	12
6	22	Female	20.5	No	No	No	15	No	No	Nil	10
7	28	Male	30	No	No	No	20	No	Unknown	Nil	10
8	40	Male	33	Yes	No	Yes	Unknown	No	Yes	5	4
9	33	Male	26.8	Yes	No	Yes	20	No	No	Nil	10
10	41	Female	39	Yes	No	Yes	30	No	Yes	5	13
11	60	Female	29	Yes	No	Yes	30	No	Yes	5	6
12	21	Male	24.6	No	No	No	21	No	Yes	5	3
13	27	Male	27.7	No	No	No	30	No	Yes	4	2
14	56	Male	N/A	Yes	No	Yes	52	No	Yes	4	2
15	42	Male	32	No	No	No	12	No	No	Nil	2
16	60	Female	N/A	Yes	No	Yes	38	No	Yes	4	9
17	40	Male	N/A	Yes	No	Yes	30	No	Yes	2	11

N/A, non-available.

**Table 3:** Affected site, presentation, MRI findings, and treatment plan for each case

No.	Site	Presentation	MRI finding	Treatment
1	Bilateral	Bilateral hip pain and limping	FHAVN Left side grade 2, right side grade 3	Bilateral core decompression+plasma injection
2	Bilateral	Bilateral hip pain, thigh pain, inability to walk distance and limping	FHAVN Left side grade 4, right side grade 4	Refused operation, conservative
3	Bilateral	Bilateral hip pain and limping	FHAVN Left side grade 4, right side grade 3	Refused operation, conservative
4	Bilateral	Bilateral hip pain, limping and inability to walk	FHAVN Left side grade 3, right side grade 3	Bilateral THR
5	Bilateral	Bilateral hip pain and thigh pain and numbness	Bilateral FHAVN	Bilateral core decompression
6	Bilateral	Bilateral hip pain	Bilateral FHAVN	Bilateral core decompression
7	Bilateral	Bilateral hip pain	Bilateral FHAVN	Bilateral core decompression
8	Bilateral	Bilateral hip pan	Bilateral FHAVN	Conservative
9	Bilateral	Bilateral hip pain	FHAVN Left side grade 4, right side grade 4	Bilateral THR
10	Right	Right hip pain	Right side FHAVN grade 2	Conservative
11	Bilateral	Bilateral hip pain	FHAVN Left side grade 2, right side grade 3	Refused operation, conservative
12	Left	Left hip pain on walking	Left side FHAVN grade 3	Refused operation, conservative
13	Bilateral	Bilateral hip pain and limping	FHAVN Left side grade 2, right side grade 2	Refused operation, conservative
14	Bilateral	Bilateral hip pan	FHAVN Left side grade 3, right side grade 3	Refused operation, conservative
15	Left	Left hip pain and limping	Left side FHAVN grade 3	Refused operation, conservative
16	Bilateral	Bilateral hip pain and limping	FHAVN Left side grade 3, right side grade 2	Bilateral core decompression
17	Bilateral	Bilateral hip pain and limping	FHAVN Left side grade 3, right side grade 3	Bilateral THR

FHAVN, femoral head avascular necrosis; THR, total hip replacement.

## Discussion

AVN is a condition in which the blood supply to the bone is disrupted, leading to bone death and subsequent collapse of the bone structure^9^. AVN of the hip occurs frequently as a result of trauma, most commonly following a femoral neck fracture or hip dislocation. Several causal links to non-traumatic AVN have been discovered^10^. In the majority of cases, non-traumatic AVN is associated with the use of alcohol, glucocorticoids, co-existing haematological disorders (such as sickle cell anaemia, thalassaemia, polycythemia, and haemophilia), myeloproliferative disorder, Gaucher disease, and conditions, such as hypercholesterolaemia, pregnancy, chronic renal failure, hyperparathyroidism, and Cushing’s disease. However, in around 30% of patients, the cause of non-traumatic AVN remains unknown, and they are classified as idiopathic. The presence of AVN in twins and family clusters suggests that genetic factors may be involved^11^. The combination of loss of blood perfusion and elevated intraosseous pressure has been recognized as the primary cause of the necrotic process^12^. The male-to-female ratio is around 4:1, with an average age of onset in the fifth decade. Bilateral AVN has been documented in more than 50% of cases^10^. In up to 72% of patients, AVN is characterized by nonspecific symptoms^13^. The mean age of patients in the current study was 38.65 years, and males made up 70.6% of the patients. Bilateral femoral head was involved in 82.3% of the cases. All of the patients had a negative history of bone disease.

Given that the long-term consequences of COVID-19 are predicted to pose a physical, psychological, and financial burden on patients, caregivers, and healthcare systems, it is critical to remember that there may be complications following recovery, including adverse non-pulmonary complications^6,14^. AVN is a rare sequela that, if not treated, can result in severe complications, including bone collapse ^6^.

Many medications that may be effective against COVID-19 have been investigated since the pandemic, including antivirals, angiotensin receptor blockers, chloroquine phosphate, and corticosteroids^15^. The RECOVERY clinical trial, one of the largest trials on COVID-19 treatments, found that corticosteroids reduced the risk of mortality in hospitalized patients with severe COVID-19 who were on a ventilator or receiving oxygen at a 20% concentration^16^. Although corticosteroids are life-saving in the treatment of COVID-19, they are also a risk factor for the development of AVN^15^. Corticosteroid-associated AVN accounts for roughly 10–30% of non-traumatic AVN which makes it the most prevalent aetiology of non-traumatic AVN^10^. As a result, corticosteroids should be used with caution, with care given to the dose and duration. Tocilizumab, a new medication, may be used instead of corticosteroids to suppress the cytokine storm^17^. It is also unclear whether the risk of AVN is more closely related to the total dosage, the maximal dose, or the duration of treatment^18^. According to some studies, AVN development requires a cumulative dosage of 2000 mg of prednisone^15^. Previous rare case reports have described individuals who developed AVN after being administered low-dose steroid therapy^19^. A study suggests that a risk classification method for the AVN in COVID-19 patients should be developed: (i) low-risk clients would receive no corticosteroids; (ii) moderate-risk clients would receive corticosteroids for less than 1 week with a cumulative dose of less than 2000 mg; and (iii) high-risk patients would receive corticosteroids for at least 1 week with a cumulative dose of 2000 mg or an intravenous pulse of 80 mg/day for at least 3 days^20^. In the current study, eleven (64.7%) patients reported receiving steroid treatments during the infection. All eleven patients received a single dosage of steroid injection for different periods like a week (3 cases), 5 days (4 cases), and 4 days or less (4 cases).

There is still uncertainty about the time interval between steroid injection and the onset of AVN symptoms. Zhao *et al*.^21^ reported an interval of up to 3 years. McKee *et al*.^22^ found a mean period of 16.6 months. Following the COVID-19 diagnosis, Agarwala *et al*.^15^ discovered a mean time of 58 days (range 45–67 days) for the development of AVN. According to Agarwala *et al*.^15^, the sensitivity to developing AVN is increased owing to the COVID-19 virus, and a lower cumulative dosage of steroids is required. The mean interval from COVID-19 infection to the presentation in the current cases was 6.53 months. Other drugs used to treat COVID-19 infection, such as lopinavir and ritonavir, can also cause osteonecrosis. Some virus-related processes have also been proposed as probable causes, including hypercoagulability caused by SARS-CoV-2 and an increase in bone resorption mediated by the ACE-2 receptor. Despite all of these risk factors, only a few cases of AVN following COVID-19 have been documented^23^.

Diagnosis of AVN in the early stage (stage I) is critical for preventing the disease’s progression and preventing bone collapse^24^. Plain radiography, MRI, computer-aided tomography, skeletal scintigraphy, and single-photon emission computed tomography are now available as noninvasive diagnostic techniques for AVN^11^. X-ray-based radiography detection is insufficient to identify AVN at its onset, and its overall sensitivity for early-stage AVN is only around 41%^25^. MRI is more useful than X-rays in identifying AVN in its early stages^26^. If not detected at early stages, this problem progresses to osteoarthritis and, eventually, joint replacement^12^. All cases of the current study were confirmed to have AVN through MRI study.

There has been a greater emphasis on early treatments for AVN^26^. A variety of non-surgical and surgical approaches have been described^27^. In the early stages of AVN, minimal weight-bearing and pharmacological agents, such as lipid-lowering medications, vasodilators, anticoagulants, and bisphosphonates may be beneficial^27^. Although CD is the most generally approved therapy for early-stage AVN of the femoral head, its usage has been criticized due to its poor effectiveness^28^. Other surgical approaches include osteotomy and bone graft which may assist in slowing the progression of the disease. If non-surgical and joint-saving techniques fail, arthroplasty may be a viable treatment option^29^. Because of the predicted long-life expectancy in young patients and the short survival time of arthroplasties, preservation of the femoral head and the hip joint is preferable and is the aim of almost all treatment options^27^. In the current study, bilateral CD was performed in five cases, three cases were scheduled to undergo total hip replacement, and nine cases received conservative therapy due to patient refusal for surgery.

Following discharge, several follow-up strategies should be implemented for COVID-19 patients according to the varied risks. The MRI is the ideal imaging technique for early diagnosis of AVN of the femoral head^18^. Bisphosphonates and vitamin E should be prescribed to patients while on corticosteroid therapy. Anticoagulants, vasodilators, and traditional Chinese medicine may also be alternatives^30^. Physical therapy and combination medication can be utilized to postpone or prevent the collapse of steroid-induced AVN of the femoral head in the early stages of the disease^31^.

In conclusion, the ongoing pandemic may have many long-term sequelae. There is a risk of developing AVN after COVID-19. Early detection of AVN is critical because the resulting bone collapse can be avoided if early treatment is started.

## Ethical approval

Ethical approval for this study (Ethical Committee No: 175) was provided by the Ethical Committee of School of Medicine-University of Sulaimani on 4 January 2021.

## Consent

Written informed consent was obtained from the patient for publication and any accompanying images. A copy of the written consent is available for review by the Editor-in-Chief of this journal on request.

## Sources of funding

The research has got no funding.

## Author contribution

S.K.A. was a major contributor to the conception of the study, as well as in the literature search for related studies, final approval of the manuscript. M.N.H. and F.H.K. were involved in writing the manuscript, literature review, final approval of the manuscript. M.S., A.M.S. and R.K.A. were involved in the literature review, the design of the study, revision of the manuscript. B.A.A.was involved in critical revision of the manuscript and final approval. R.Q.S. and S.H.M. confirm the authenticity of all the raw data, revision of the manuscript

## Conflicts of interest disclosure

The authors declare that they have no conflicts of interest.

## Research registration unique identifying number (UIN)

The research was registered in the Research Registry with a registration number ofresearchregistry7468.

https://www.researchregistry.com/register-now#userresearchregistry/registerresearchdetails/61c087e7d880dc001ecc84a9/

## Guarantor

Fahmi Hussein Kakamad.

## Data availability statement

Not applicable.

## Provenance and peer review

Not commissioned, externally peer-reviewed.
